# Dynamic vehicle routing with time windows in theory and practice

**DOI:** 10.1007/s11047-016-9550-9

**Published:** 2016-04-09

**Authors:** Zhiwei Yang, Jan-Paul van Osta, Barry van Veen, Rick van Krevelen, Richard van Klaveren, Andries Stam, Joost Kok, Thomas Bäck, Michael Emmerich

**Affiliations:** 1 Leiden Institute of Advanced Computer Science, Leiden University, Niels Bohrweg 1, 2333 CA Leiden, The Netherlands; 2 School of Information System and Management, National University of Defense Technology, Changsha, 410073 Hunan People’s Republic of China; 3 Almende, Westerstraat 50, 3016 DJ Rotterdam, The Netherlands

**Keywords:** Ant colony optimization, Vehicle routing problem, Dynamic vehicle routing problem with time windows, Pilot study

## Abstract

The vehicle routing problem is a classical combinatorial optimization problem. This work is about a variant of the vehicle routing problem with dynamically changing orders and time windows. In real-world applications often the demands change during operation time. New orders occur and others are canceled. In this case new schedules need to be generated on-the-fly. Online optimization algorithms for dynamical vehicle routing address this problem but so far they do not consider time windows. Moreover, to match the scenarios found in real-world problems adaptations of benchmarks are required. In this paper, a practical problem is modeled based on the procedure of daily routing of a delivery company. New orders by customers are introduced dynamically during the working day and need to be integrated into the schedule. A multiple ant colony algorithm combined with powerful local search procedures is proposed to solve the dynamic vehicle routing problem with time windows. The performance is tested on a new benchmark based on simulations of a working day. The problems are taken from Solomon’s benchmarks but a certain percentage of the orders are only revealed to the algorithm during operation time. Different versions of the MACS algorithm are tested and a high performing variant is identified. Finally, the algorithm is tested in situ: In a field study, the algorithm schedules a fleet of cars for a surveillance company. We compare the performance of the algorithm to that of the procedure used by the company and we summarize insights gained from the implementation of the real-world study. The results show that the multiple ant colony algorithm can get a much better solution on the academic benchmark problem and also can be integrated in a real-world environment.

## Introduction

The vehicle routing problem (VRP) is a combinatorial optimization problem which has been studied for a long time in the literatures, such as Bianchi et al. (2009), Marinakis et al. (2010), Xiao et al. (2012), Pillac et al. (2013) and Yang et al. (2015). The aim of this problem is to deliver orders from depot to customers using a fleet of vehicles. Here we look at a practically important variant of this problem where new events (demands, orders) are dynamically introduced during operation time and cars have to serve customers at times within given time windows. So far the problems of dynamical events and time windows have only been looked at in isolation, but in this paper we will propose and analyze an algorithm that can deal with dynamicity and time windows.

Since the VRP problem already in its most basic variant is *NP* hard it seems unlikely that efficient exact solvers for larger instances can be built and one has to rely on heuristics and meta-heuristics for finding good solutions. Among these heuristic methods, problem specific heuristics, including savings heuristic, local search meta-heuristics, and approaches from natural computing such as ant colony optimization are common approaches for solving this problem. Yet, the most powerful solvers today combine several of these methods and could be termed *hybrid solvers*.

In this article a hybrid solver is developed. In the global search architecture it uses an ant colony optimization system, whereas in its initialization and search operators it uses problem specific construction and local search methods. More specifically, the multi ant colony system (MACS) is introduced to solve the real-world dynamic vehicle routing problem. MACS was first proposed by Gambardella et al. (1999) which used two ant colonies to search the best solution for the vehicle routing problem in order to improve the performance of ant colonies. In this algorithm, the first colony minimizes the number of vehicles while the second one minimizes the travel cost. van Veen et al. (2013) generate a dynamic vehicle routing problem with time windows (DVRPTW) benchmark based on the static Solomon benchmark and adjust the MACS to this dynamic problem. This article extends upon this conference paper by providing a more in-depth discussion and motivation of the approach and benchmark designs. More importantly, we add results from a real-world pilot study provided by a Dutch mobile surveillance company.

This paper is organized as follows: The problem is formally described in Sect. 2. Related work is summarized in Sect. 3. Section 4 describes the MACS algorithm and how it is adapted to the dynamical vehicle routing problem with time windows. Section 5 introduces a benchmark for this problem class and describes the performance of the algorithm on the benchmark and also includes results on static benchmarks for validation. The real-world study, set up in Rotterdam, is described in Sect. 6 and we summarize the experiences gained from the case study. Section 7 reviews the main results of this article. Finally, Sect. 8 summarizes the work of this article and suggests directions for relevant future research.

## Problem description

### Static vehicle routing problem

The classical VRP formulation was first defined by Dantzig and Ramser (1959). In classical VRP, a fleet of vehicles seek to visit all orders of the customers at minimum travel cost. This problem is an NP-hard problem and the well known traveling salesman problem (TSP) is a special case. Next, we will look at the capacitated VRP (CVRP), where each vehicle has a maximal capacity. It can be modeled by introducing a weighted digraph $G=(V, A)$, where $V=\{v_0, v_1, \ldots , v_N\}$ is a vertex set representing the customers and $A=\{(v_i, v_j); i \ne j \}$ is an arc set, where $(v_i, v_j)$ represents the path from customer *i* to customer *j*. Vertex $v_0$ represents the depot which has *M* vehicles, and vertices ($v_1, \ldots , v_M$) denote the customers that need to be served. Each vehicle has a maximal capacity *Q* and each customer $v_i$ is associated with a demand $q_i$ of goods to be delivered (the demand $q_0 = 0$ is associated to the depot $v_0$), a time window $[e_i, l_i]$ from the earliest starting time to the latest starting time for the service, and the duration (time) of a service $s_i$. Each arc $(v_i,v_j)$ has a non-negative value weight representing its traveling cost $c_{ij}$. There are *N* customers and *M* vehicles. The goal is to minimize the traveling cost.

Formally, the CVRP can be defined as a mathematical programming problem with binary decision variables (cf. Christofides et al. 1981; Cordeau et al. 2001). Let $\xi _{ijk}=1$, if vehicle *k* visits customer $x_j$ immediately after visiting customer $x_i$, and $\xi _{ijk}=0$ otherwise. Now, the mathematical programming problem reads:

1 $$\text {minimize} \,z=\sum _{i=0}^{N}\sum _{j=0}^{N} \left( c_{ij}\sum _{k=1}^{M}\xi _{ijk}\right),$$

subject to

2a $$\sum _{i=0}^{N}\sum _{k=1}^{M}\xi _{ijk}=1, \quad j=1,\ldots , N,$$

2b $$\sum _{i=0}^{N}\xi _{ipk}-\sum _{j=0}^{N}\xi _{pjk}=0, \quad k=1,\ldots ,M, \; p=0,\ldots ,N,$$

2c $$\sum _{i=1}^{N}\left( q_i\sum _{j=0}^{N}\xi _{ijk}\right) \le Q, \quad k=1,\ldots ,M,$$

2d $$\sum _{i=0}^{N}\sum _{j=0}^{N}c_{ij}\xi _{ijk}+\sum _{i=1}^{N}\left( s_i\sum _{j=0}^{N}\xi _{ijk}\right) \le T, \quad k=1,\ldots ,M,$$

2e $$\begin{aligned}&\sum _{j=1}^{N}\xi _{0jk}=1, \quad k=1,\ldots ,M, \\&\xi _{ijk} \in \{0,1\} \quad \text{ for } \text{ all } \text{i, j, k } \end{aligned}$$

Here, the constraints of the formulation can be explained as the constraints of VRPs. In detail the constraint equations above are motivated as follows.

Eq. 2a: Each customer must be visited exactly once.
Eq. 2b: If a vehicle visits a customer, it must also depart from it.
Eq. 2c: The total quantity in each vehicle is less or equal to the maximal capacity *Q*.
Eq. 2d: The total traveling time of each vehicle is less or equal to a given time T.
Eq. 2e: Each vehicle must be used exactly once.

In this work we are going to consider the vehicle routing problem *with time windows* in which to serve the customers (CVRPTW). Additional constraints are needed for modeling time windows. In this case the start serving time $t_i$ to vertex $v_i$ is between the time windows $[e_i, l_i]$.

### Dynamic vehicle routing problem

In the real world, most of the delivery problems are dynamic vehicle routing problems. Psaraftis (1995) pointed out the difference between static VRPs and dynamic VRPs. In the static VRPs, the information of the orders is known in advance. While in dynamic problems, some of the orders are given initially and an initial schedule is generated. But new orders are dynamically received when the vehicles have started executing the routes and the route has to be rearranged in order to serve these new orders. The challenge is whether the algorithm can give a high quality solution quickly when the new event happens.

To be able to solve a *dynamic problem* we first have to simulate a form of dynamicity. Kilby et al. (1998) have described a method to do this, which is also used by Montemanni et al. (2005). They proposed to partition the working day into time slices and solve problems incrementally. The notion of a working day of $T_{wd}$ seconds is introduced, which will be simulated by the algorithm. Not all nodes are available to the algorithm at the beginning. A subset of all nodes are given an *available time* at which they will become available. This percentage determines the degree of dynamicity of the problem. At the beginning of the day a tentative tour is created with a-priori available nodes. The working day is divided into $n_{ts}$ time slices of length $t_{ts} := T_{wd}{/}n_{ts}$. At each time slice the solution is updated. This allows us to split up the dynamic problem into $n_{ts}$ static problems, which can be solved consecutively. The goal in DVRPTW is similar to that of static VRPs, except that some customers and their time windows are unknown a-priori and parts of the solutions might already have been committed.

In our approach the previous solution and the pheromone distribution of the ant colony optimization algorithm is used as initialization to the optimization in a time slice, because we expect the new solution not to be entirely different from the previous one. A different approach would be to restart the algorithm from scratch every time a node becomes available. However, this strategy is too time consuming for algorithms used in real time operation and on typical hardware used by logistics service providers.

## Related work

In general VRP and VRPTW are NP hard problems and they generalize the NP-complete traveling salesman problem. Therefore heuristic algorithms are widely used in order to solve the vehicle routing problem. Classical examples are the *nearest neighbor heuristic* by Flood (1956) and the *savings algorithm* that was developed by Clarke and Wright (1964) based on the savings concept which repeatedly combines two customers on the same route. Early advances were achieved by Shaw (1998) using large neighborhood search.

Nowadays, the use of meta-heuristics becomes more and more popular. Semet and Taillard (1993) presented a tabu search for finding a good solution for the vehicle routing problem. Baker and Ayechew (2003) combined the genetic algorithm and neighborhood search methods which can give a reasonable results for this problem. Gambardella et al. (1999) introduced ant colony optimization which can use artificial ant colonies to construct a shortest route.

In contrast to a large multitude of available static VRP solvers, there are only a few algorithms which can tackle dynamic VRPs. In principle, most of the algorithms described above can be adapted to solve the dynamic VRPs. But in order to deal efficiently with the dynamics of this problem, the algorithm should also have some mechanisms that promote reusing learned features of the problem from previous solutions. As indicated in Eyckelhof and Snoek (2002), some bio-mimetic ant-colony optimization algorithm seems to support dynamic adaptations of delivery routes well. For instance, in ant colony optimization virtual pheromone trails are created to indicate good directions if solutions only need to be changed partially.

Ant colony optimization (ACO) is a meta-heuristic algorithm based on the natural behavior of the ant colony which was proposed by Dorigo (1992) in his Ph.D. thesis. More recently, it has been employed in a number of combinatorial optimization problems, such as scheduling problems in Xiao et al. (2013), Chen and Zhang (2013), routing problems in Balaprakash et al. (2009), Toth and Vigo (2014), assignment problems in Dorigo and Stützle (2010), D’Acierno et al. (2012), set problems in Ren et al. (2010), Jovanovic and Tuba (2013) and so on. Moreover, ACO can be easily combined with local search heuristics and route construction algorithms. The flexibility of ACO and its good performance in static vehicle routing problem make it an attractive paradigm for the dynamic vehicle routing problem.

Ant-based methods were first proposed with the ant system method in Colorni et al. (1991). These methods simulate a population of ants which use pheromones to communicate with each other and collectively are able to solve complex path-finding problems—a phenomenon called *stigmergy*. For the VRPTW problem, an ant-based method was proposed by Gambardella et al. (1999). They showed that good results can be achieved by running one ant colony for optimizing the number of vehicles and one ant colony for minimizing route cost and term their method multi ant colony system (MACS). The paradigm of ant algorithms fits well to dynamic problems in Guntsch and Middendorf (2002) including TSP in Eyckelhof and Snoek (2002) and special types of VRP problem, where vehicles do not have to return to the depot which can be seen in Montemanni et al. (2005). In our article we will extend multi ant colony optimization to problems *with time windows* and we will call our new method MACS-DVRPTW.

There exist some previous studies on using meta-heuristics other than ant colony algorithms on DVRPTW. Gendreau et al. (1999) propose to use tabu search, but, as opposed to standard benchmarks for MACS-VRPTW, developed their approach for problems with soft time windows.

## Algorithm

In order to solve this problem, it is natural to extend the state-of-the-art ant algorithm for VRPTW to the dynamical case. To our best knowledge, the multi-colony approach described in Gambardella et al. (1999) is the best ant algorithm for the VRPTW with a description that allows to reproduce results, and it shows a good performance on standard benchmark problems by Solomon. Here we will directly describe our new dynamic version of this algorithm and indicate changes.

The central part of the algorithm is the *controller*. It reads the benchmark data, initializes data structures, builds an initial solution and starts the ACS-TIME colony and ACS-VEI colony. The ACS-TIME colony tries to minimize traveling cost given a fixed number of vehicles, the ACS-VEI colony seeks to minimize the number of vehicles. Priority of the algorithm is on reducing the number of vehicles. Given solutions with the same number of vehicles, those solutions are preferred that use less time. The ACS-VEI colony restarts the ACS-TIME colony whenever a solution is found that can serve the demand with a smaller number of vehicles.

The *nearest neighbor heuristic* in Flood (1956) is used to find initial solutions of vehicle routing problems. But for the VRPs with time windows, it is difficult to get a feasible solution by using this method. So it has to be adjusted in two ways. First the constraints on time windows have to be checked to make sure no infeasible tours are created. Besides, a limit on the number of vehicles is passed to the function. Therefore, a more appropriate algorithm is needed to generate the initial solution. Because of these limitations, it is not always possible to return a tour that incorporates all nodes. In that case a tour with less nodes is returned.

The new initial Ranking Time Windows Based Nearest Neighbor algorithm is proposed to generate the initial solution for the DVRPTW. By adding the sorted earliest arrival time of the orders to exact $n_v$ tours one by one, this algorithm can take the time windows and vehicles number constrains in advance. This way there is a higher chance to get a feasible solution with better fitness value. Algorithm 1 describes the initialization. It proceeds as follows: Firstly, the list of customers is sorted by increasing values of earliest arrive times. Then, $n_v$ tours are created, each of which corresponds to one vehicle. For each customer node find the tour with smallest distance among all those tours in which the node can be inserted without violating constraints. Following this procedure, the nodes are iteratively added in the node list. Finally, the resulting solution is returned.

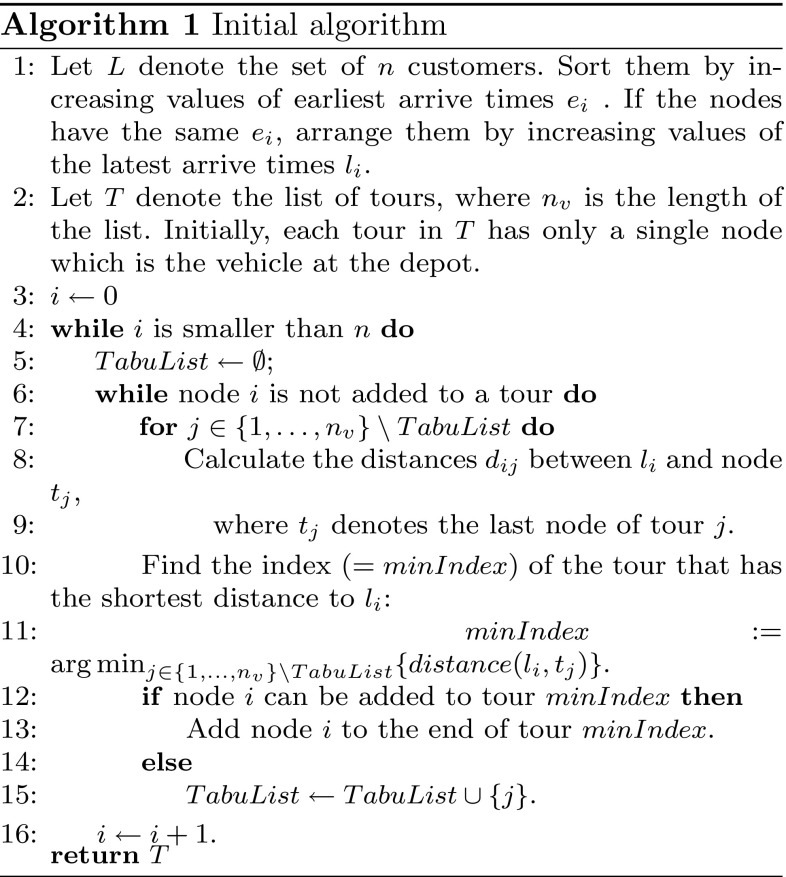



After initialization, a timer is started that keeps track of *t*, the used CPU time in seconds. Then the algorithm will run on line during the working day which ends at some point in time denoted with $T_{wd}$. Let $T^*$ denote the currently optimal solution. Then, at the start of each time slice the controller checks if any new customer nodes became available during the last time slice. If so, these new nodes are inserted using the *InsertMissingNodes* method, in order to update $T^*$. Thereafter, some of the nodes are changed to the status *committed*. The position of committed nodes in the tour cannot be changed anymore. If $v_i$ is the last committed node of a vehicle in the tentative solution, $v_j$ is the next node and $t_{ij}$ is travel time from node $v_i$ to node $v_j$, then $v_j$ is committed if $e_j - t_{ij} < t + t_{ts}$. When the necessary commitments have been made the two ant colony systems (ACS) are started. If a new time slice starts, the colonies are stopped and the controller repeats its loop.

The pseudo-code of the controller can be seen in Algorithm 2. ACS contains two colonies, each one of which tries to improve on a different objective of the problem. The ACS-VEI colony searches for a solution that uses less vehicles than $T^*$. The ACS-TIME colony searches for a solution with a smaller traveling cost than the cost in $T^*$ while using at most as many vehicles as the best solution so far, i.e. $T^*$. A solution with less vehicles has a higher priority than a solution with a smaller distance. Once a feasible solution is found by ACS-VEI, the controller restarts.

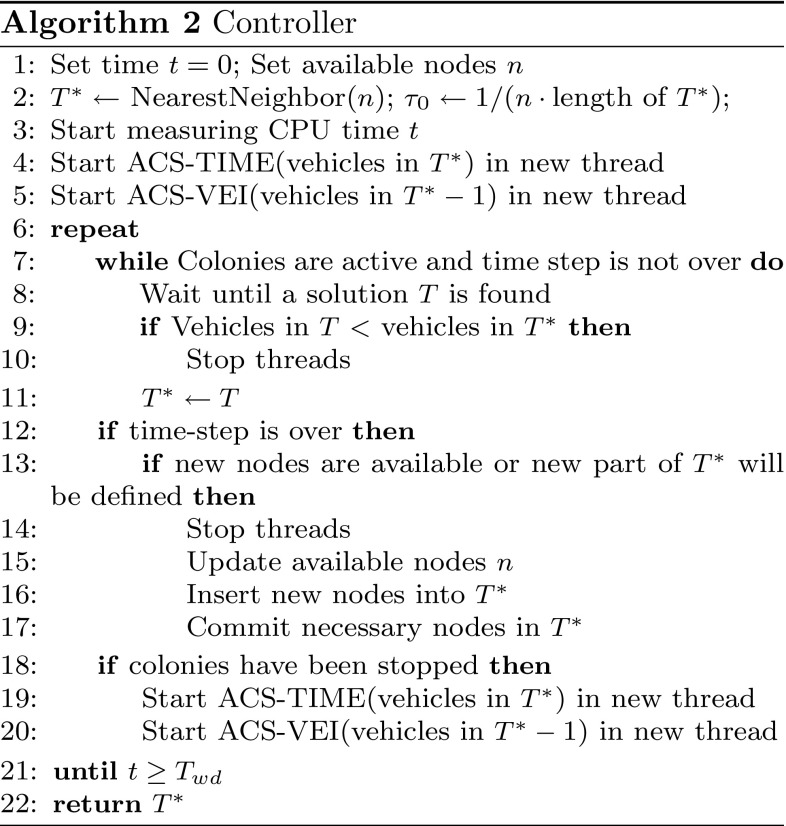



**Fig. 1:**
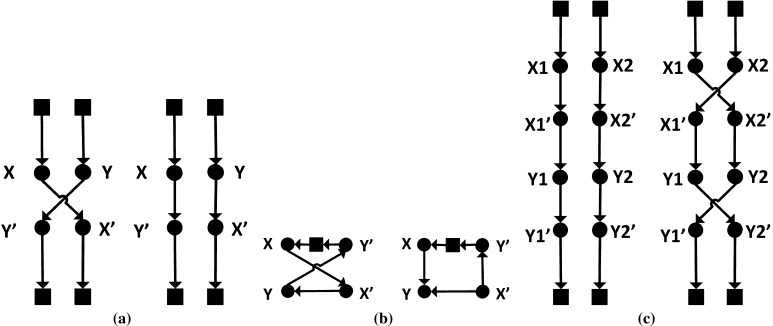
Examples of 2-opt edge replacements. *Squares* represent depots, *circles* represent nodes. **a** Demonstrates a move with edges from different tours. **b** Is an example of a move within a single tour. **c** Shows the process of cross exchange

There are a few differences between the two colonies. ACS-VEI keeps track of the best solution found by the colony ($T^{\mathrm{VEI}}$), which does not necessarily incorporate all nodes. As $T^{\mathrm{VEI}}$ also contributes to the pheromone trails it helps ACS-VEI to find a solution that covers all nodes with less vehicles. ACS-VEI does not use local search methods. In contrast, ACS-TIME does not work with infeasible solutions and it performs a local search method called *Cross Exchange* in Taillard et al. (1997) which is shown in Fig. 1.

A constraint on the maximum number of vehicles that can be used is given as an argument to each colony. During the construction of a tour this number may not be exceeded. This may lead to infeasible solutions that do not incorporate all nodes. If a solution is not feasible it can never be send to the controller. Both colonies work on separate pheromone matrices and send their best solutions to the controller. Pseudo-codes for ACS-VEI and ACS-TIME can be found in Algorithm 3 and 4, respectively.

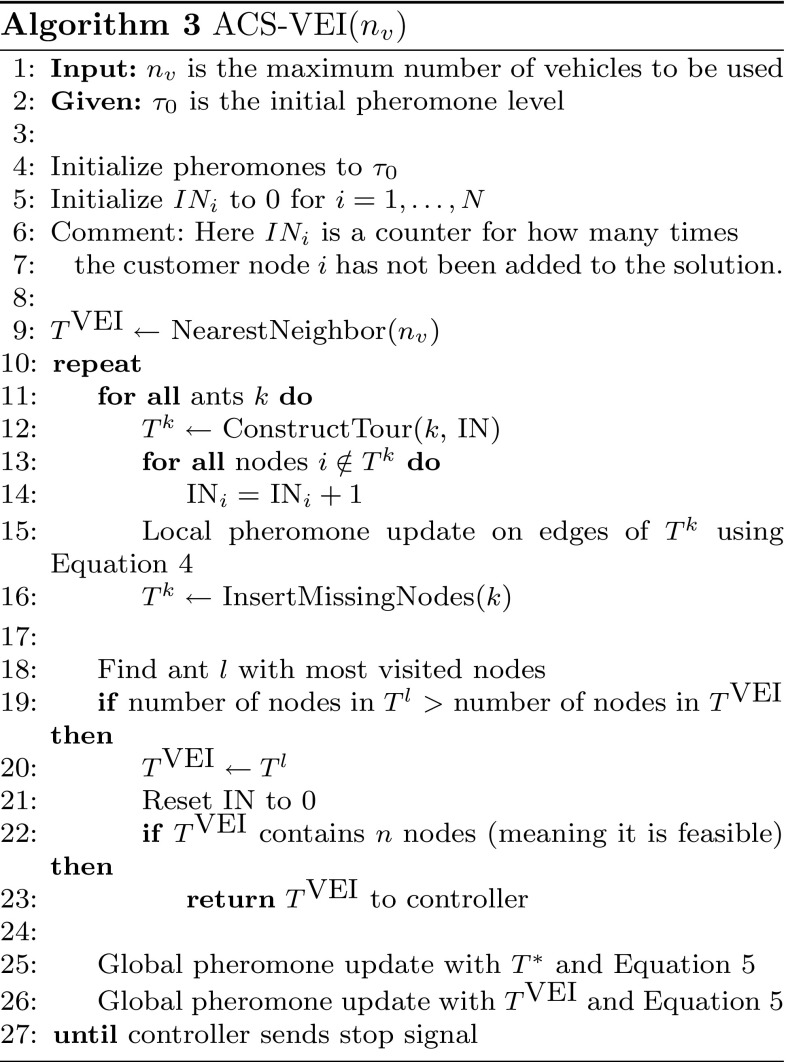

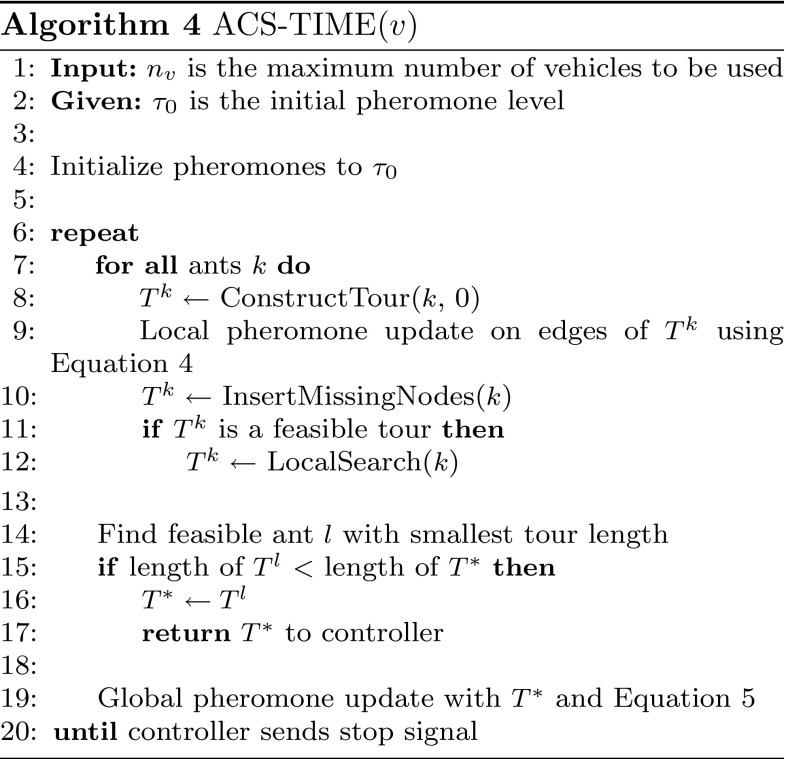





Algorithm 5 describes the *construction of a tour* by means of artificial ants. A tour starts at a randomly chosen depot copy. When constructing a new tour, the committed parts of $T^*$ which cannot be changed any more have to be incorporated first. Then the tour is iteratively extended with available neighborhood nodes. There are many ways to define the topology structure of neighborhood nodes. In the paper, the neighborhood nodes are defined as all the available nodes that have not been committed and visited yet. The neighborhood nodes set ${\mathcal{N}}^k_i$ contains all available nodes which have not been committed and visited for ant *k* situated at node *i*. Inaccessible nodes due to capacity or time window constraints are excluded from ${\mathcal{N}}^k_i$. In order to decide which node to chose, the probabilistic transition rules by Dorigo and Gambardella (1997) are applied. For ant *k* positioned at node $v_i$, the probability $p_{j}^{k}(v_i)$ of choosing $v_j$ as its next node is given by the following transition rule:

3 $$p^{k}_{j}(v_i) = {\left\{ \begin{array}{ll} \underset{j \in {\mathcal{N}}_i}{\arg \max } \{[\tau _{ij}]^{\alpha } \cdot [\eta _{ij}]^{\beta }\} &{} \text{ if } \,q \le q_0 \text{ and } j \in {\mathcal{N}}^k_i \\ \dfrac{[\tau _{ij}]^{\alpha } \cdot [\eta _{ij}]^{\beta }}{\sum _{m \in {\mathcal{N}}^k_i}[\tau _{im}]^{\alpha } \cdot [\eta _{im}]^{\beta }} &{} \text{ if } \,q > q_0 \text{ and } j \in {\mathcal{N}}^k_i\\ &{} \\ 0 &{} \text{ if } \,j \not \in {\mathcal{N}}^k_i \end{array}\right.}$$

with $\tau _{ij}$ being the pheromone level on edge (*i*, *j*), $\eta _{ij}$ the heuristic desirability of edge (*i*, *j*), $\alpha$ the influence of $\tau$ on the probabilistic value, $\beta$ the influence of $\eta$ on the probabilistic value, ${\mathcal{N}}^k_i$ the set of nodes that can be visited by ant *k* positioned at node $v_i$, and $\tau _{ij}, \eta _{ij}, \alpha , \beta \ge 0$. Moreover *q* denotes a random number between 0 and 1 and $q_0 \in [0,1]$ a threshold.

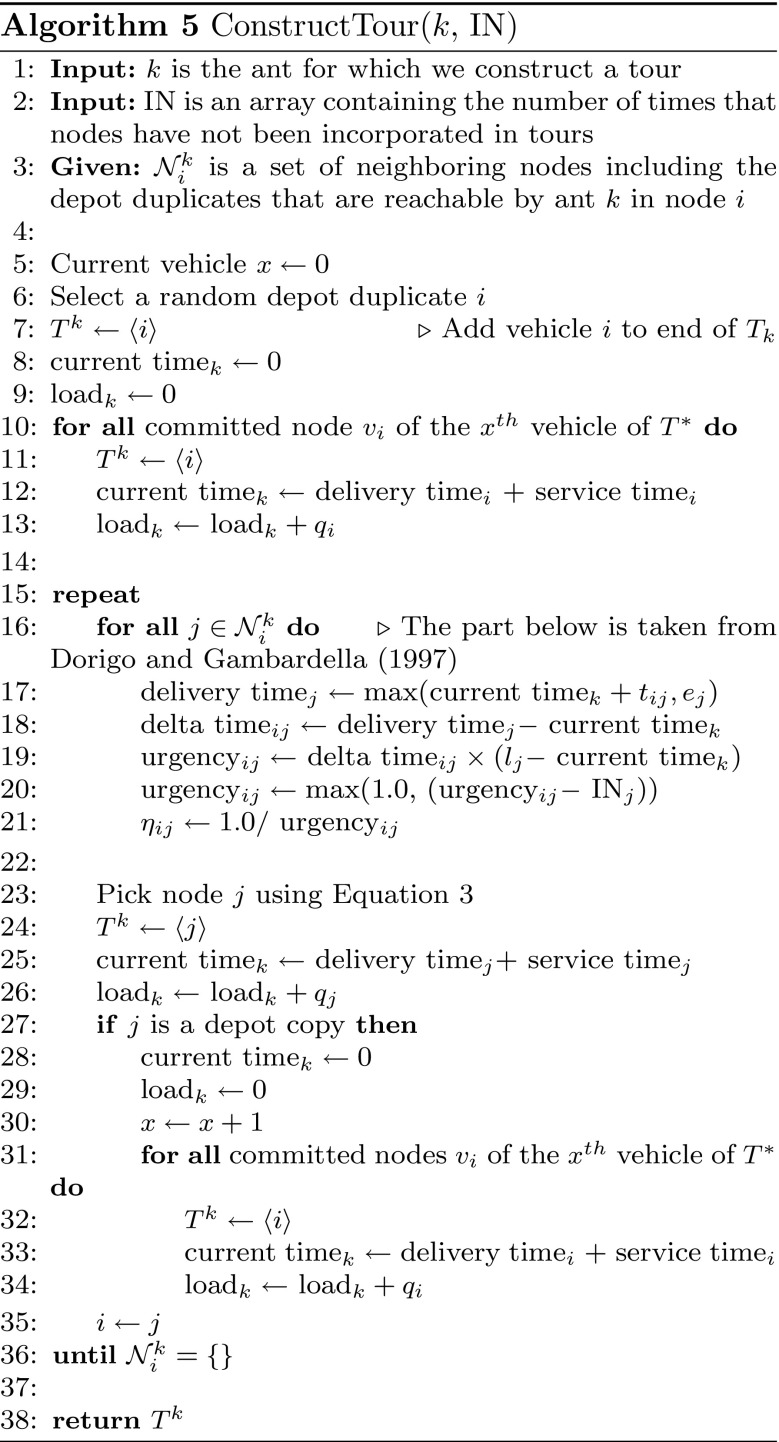



During the ConstructTour process of ACS-VEI, the IN array is used to give greater priority to nodes that are not included in previously generated tours. The array counts the successive number of times that node $v_j$ was not incorporated in constructed solutions. This count is then used to increase the attractiveness $\eta _{ij}$. The IN array is only available to ACS-VEI and is reset when the colony is restarted or when it finds a solution that improves $T^{\mathrm{VEI}}$. ACS-TIME does not use the IN array, which is equal to setting all values in the array to zero.

The local pheromone update rule from Dorigo and Gambardella (1997) is used to decrease pheromone levels on edges that are traversed by ants and it will be briefly described next. Each time an ant has traversed an edge (*i*, *j*), it applies Eq. (4).

4 $$\tau _{ij} = (1 - \rho ) \cdot \tau _{ij} + \rho \cdot \tau _0$$

By decreasing pheromones on edges that are already traveled on, there is a bigger chance that other ants will use different edges. This increases exploration and should avoid too early stagnation of the search.

The global pheromone update rule is given in Eq. (5). To increase exploitation, pheromones are only evaporated and deposited on edges that belong to the best solution found so far and $\Delta \tau _{ij}$ is multiplied by the pheromone decay parameter $\rho$.

5 $$\begin{aligned}&\tau _{ij} = (1 - \rho ) \cdot \tau _{ij} + \rho \cdot \sum _{k=1}^{m} \Delta \tau _{ij}^{k} \; ,\forall (i,j) \in T^* \\&\quad \hbox { and }\Delta \tau _{ij}^{k} = 1 / L^* \end{aligned}$$

where $T^*$ is the best tour found so far and $L^*$ is the length of $T^*$.

Gambardella et al. (1999) has shown that the MACS is very efficient in solving *static* vehicle routing problems with time windows. Here we are going to test and benchmark the extended algorithm for *dynamic* vehicle routing problems with time windows.

## Benchmark on simulated data

The Solomon benchmark is a classical benmark for static VRP in Solomon (1987). It provides 6 categories of scalable VRPTW problems: C1, C2, R1, R2, RC1 and RC2. The C stands for problems with clustered nodes, the R problems have randomly placed nodes and RC problems have both. In problems of type 1, only a few nodes can be serviced by a single vehicle. But in problems of type 2, many nodes can be serviced by the same vehicle.

In order to make this a dynamic problem set we apply a method proposed by Gendreau et al. (1999) for a VRP problem, to the more comprehensive benchmark by Solomon on VRPTW. A certain percentage of nodes is only revealed during the working day. A dynamicity of *X*% means that each node has a probability of *X*% to get a non-zero available time. The available time means the time when the order is revealed. It is generated on the interval $[0, \overline{e_i}]$, where $\overline{e_i}= \min (e_i, t_{i-1})$. Here, $t_{i-1}$ is the departure time from $v_i$’s predecessor in the best known solution. These best solutions are taken from the results of a static MACS-VRPTW implementation (see Table 1)—for the detailed schedules we refer to the support material available on http://natcomp.liacs.nl/index.php?page=code. By generating available times on this interval, optimal solution can still be attained, enabling comparisons with MACS-VRPTW. Table 2 shows the average results and standard deviation change with the dynamicity levels.

**Table 1:** Comparison of results reported for the original MACS-VRPTW in Gambardella et al. (1999) and our implementation for the Solomon benchmark

	Gambardella	Avg	Best
C1			
Dist	828.40	828.67	828.37
Vei	10.00	10.00	10.00
C2			
Dist	593.19	591.00	589.85
Vei	3.00	3.00	3.00
R1			
Dist	1214.80	1226.05	1216.70
Vei	12.55	12.52	12.33
R2			
Dist	971.97	992.49	949.69
Vei	3.05	3.00	3.00
RC1			
Dist	1395.47	1381.20	1362.58
Vei	12.46	12.25	12.00
RC2			
Dist	1191.87	1165.51	1146.89
Vei	3.38	3.35	3.25

**Table 2:** Average results and standard deviations (SD) for 10 runs and 56 problems of different MACS-DVRPTW variants and dynamicity levels (Dyn)

Dyn	0 %	10 %	20 %	30 %	40 %	50 %
Normal						
Vei	7.39	7.91	8.37	8.79	9.03	9.32
Dist	1046.06	1095.1	1131	1180.36	1217	1241.32
SD	21.72	28.95	29.59	34.84	36.73	38.09
IIS						
Vei	7.35	7.93	8.38	8.78	9.02	9.36
Dist	1035.86	1087.06	1131	1177.96	1212	1236.36
SD	20.14	28.39	31.13	34.37	37.12	39.64
WPP						
Vei	7.35	7.93	8.39	8.79	9.04	9.34
Dist	1043.13	1087.98	1128	1175.14	1210	1235.9
SD	20.22	26.11	26.52	35.32	37.80	38.52
MMAS						
Vei	7.40	7.95	8.43	8.88	9.08	9.34
Dist	1050.06	1093.66	1134	1183.02	1212	1235.9
SD	22.29	31.66	36.00	34.59	39.64	39.06

The implementation was executed ten runs on a Intel Core i5, 3.2 GHz CPU with 4 GB of RAM memory. The controller stops after 100 s of CPU time. The following default parameters are set according to the literature: $m = 10$, $\alpha = 1$, $\beta = 1$, $q_0 = 0.9$, $\rho = 0.1$ (cf. Gambardella et al. 1999), $T_{wd} = 100$ s, and $n_{ts} = 50$ (cf. Montemanni et al. 2005).

To the best of our knowledge, there is no other algorithms which have been implemented to solve this problem. In this paper, four variants of the algorithm are generated in order to improve the performance of the algorithm. Four variants of the algorithms were as follows: (1) default settings as described above, (2) spending 20 CPU seconds before the starting of the working day to construct an improved initial solution (IIS), (3) with pheromone preservation (WPP) in Montemanni et al. (2005) ($\tau _{ij}= \tau _{ij}^{old} (1-\rho ) + \rho \tau _0$), $\rho =0.3$, and (4) min–max pheromone update in Stützle and Hoos (1997). For MMAS, we set $\rho =0.8$. The values used are: $\tau _{max} = 1 / (\rho T^*)$, $\tau _{min} = \tau _{max} / (2 \cdot \text{ AvailableNodes })$, $\tau _0 = \tau _{max}$. These are updated every time a new improvement of $T^*$ is found.

Average results for IIS and MMAS are almost identical to the original results. The reason for this seems to be that although the initial solution is greatly improved, it is more difficult to insert new nodes into the current best solution. Tables 3 and 4 show results for different types of problems in more detail. WPP improves distance results for 10 % dynamicity and MMAS for 50 % dynamicity, both for the price of slightly more vehicles. Another finding is that for 10 % dynamicity solution quality declines by up to 20 % and for 50 % by up to 50 %.

From a practical approach it can be stated that for a small dynamicity of 10 % at most 1 additional vehicle is needed as compared to scheduling the same amount of static orders, and in many cases the same number of vehicles suffice. For 50 % dynamicity the number of vehicles increases almost always by one vehicle and can in some cases even increase by two vehicles.

**Table 3:** Averaged results of six Solomon categories using different variants in 10 % dynamicity

10 %	Static	DVRP, default	DVRP, 0.3 WPP	DVRP, IIS	DVRP, MMAS	Decline (%)
C1						
Dist	828.67	944.10	947.04	**943.10**	954.55	13.81
Vei	10.00	10.85	**10.87**	10.88	10.87	8.50
C2						
Dist	591.00	632.80	629.20	**628.28**	632.31	6.31
Vei	3.00	**3.67**	3.67	3.68	3.68	22.33
R1						
Dist	1226.05	1282.79	1270.34	**1267.84**	1283.23	3.41
Vei	12.52	**13.10**	13.17	13.19	13.25	4.63
R2						
Dist	992.49	1038.10	1023.40	1022.65	**1013.80**	2.15
Vei	3.00	**3.52**	3.55	3.54	3.54	17.33
RC1						
Dist	1381.20	1450.76	**1438.17**	1446.80	1458.08	4.12
Vei	12.25	**12.75**	12.80	12.80	12.82	4.08
RC2						
Dist	1165.51	1222.05	1219.73	**1213.70**	1219.99	4.13
Vei	3.35	3.61	3.56	**3.51**	3.57	4.78

The bold font is for the best for each problem

**Table 4:** Averaged results of six Solomon categories using different variants in 50 % dynamicity

50 %	Static	DVRP, default	DVRP, 0.3 WPP	DVRP, IIS	DVRP, MMAS	Decline (%)
C1						
Dist	828.67	1175.86	**1166.81**	1167.09	1179.03	40.81
Vei	10.00	**12.31**	12.46	12.48	12.40	23.10
C2						
Dist	591.00	756.48	761.60	751.26	**740.36**	25.27
Vei	3.00	4.92	4.96	4.91	**4.87**	62.33
R1						
Dist	1226.05	1367.20	**1361.35**	1364.57	1378.01	11.04
Vei	12.52	14.33	**14.25**	14.35	14.42	13.82
R2						
Dist	992.49	1146.55	1138.83	1145.02	**1111.33**	11.97
Vei	3.00	4.53	4.50	**4.46**	4.62	48.67
RC1						
Dist	1381.20	1581.72	**1571.06**	1580.63	1586.22	13.75
Vei	12.25	14.26	**14.21**	14.23	14.37	16.00
RC2						
Dist	1165.51	1420.15	1415.77	1409.61	**1386.35**	18.95
Vei	3.35	**5.60**	5.70	5.73	5.78	67.16

The bold font is for the best for each problem

## Case study

This section will explain the details of the case study. First the test case which was used for the pilots will be discussed. Then the initially implemented algorithm is described. Finally, the execution of real-world pilots will be discussed, including the intermediate revisions of the algorithms that were motivated by problems encountered in real-world testing.

### Test case

To show that the method can be successfully applied in practice, a field study (with real drivers and vehicles) was conducted. The pilot study was carried out with the Dutch security company Trigion (http://trigion.nl) on a scenario that resembles a typical working day in mobile surveillance. Every day this security company has between 300 and 400 planned jobs in the Rotterdam area. These planned jobs include surveillance, security checks, and the opening or closing of buildings, among others. There are strict contracts about the time windows and tasks which are included in such a job. Also, the average service time for each job is known. The deviation, along with a typical minimum and maximum service time is also well-known. These numbers are all derived from historical data. There is an average of about 45 incidents (or alarms) per day within the same region. However, this amount can vary from 30 to 110 incidents. These incidents can for instance be fire alarms, burglary alarms or technical problems. They appear during the day and cannot be predicted. Some predictions can be made, i.e. most alarms occur in the evening and on industrial terrains, but their exact times and other properties are not known beforehand. Therefore, this business case is perfect for implementing a DVRPTW. This DVRPTW has an average dynamicity of 11.6 %.

**Fig. 2:**
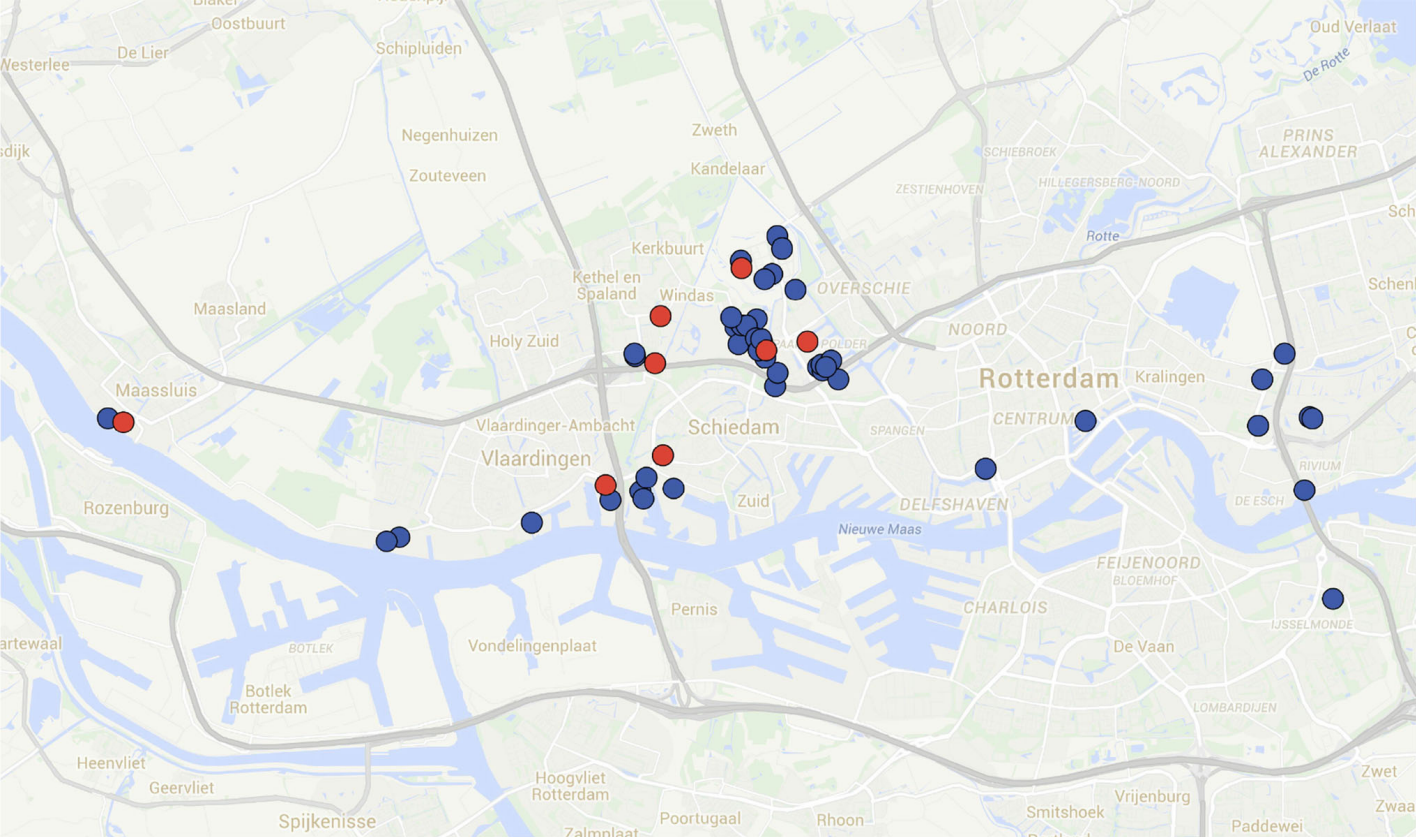
All jobs of the pilot study displayed on a map. *Blue* static jobs. *Red* incidents

To use the business case as a practical real-world testing case for a DVRPTW algorithm, the case needed to be scaled down. For 400 incidents a few dozens of vehicles would be needed. A pilot of this size would be outside of our scope, because of finances, time and complexity. Therefore, a test case of five vehicles was created with four vehicles for static jobs from the same depot and the same day. All the jobs have addresses close to each other. This resembles the problem for a smaller area with a single depot. These 4 vehicles had to cover a total workload of 48 jobs. Also, one incident vehicle from the same area and day was selected, covering nine incidents. This gives us a dynamicity of 15.8 %, $(9/(48 + 9))$ which is relatively high compared to the average of 11.6 % in the real-world business case. This was done on purpose to make a challenging test case. The 57 orders were made anonymous by selecting an address up to two streets away from the initial address. Due to the small perturbation radius this still makes a realistic test case. The time windows of the jobs within the test case all took place within a 6 h time-frame, in the evening. To get a general view of the addresses in the test case, the map with all customers is shown in Fig. 2. A characteristic of this problem is that the concentration of orders is concentrated higher in two central parts than in peripheral parts of the urban agglomeration.

In the pilot study each customer (or job) *i* has the following properties:

- A location. This is an address. The travel time, cost or distance $d_{ij}$ between two jobs *i* and *j* can be calculated by a navigation (web)service, such as Google Maps.
- A service time $s_i$. The time it takes to complete the job. The service time is not always known a-priori. Sometimes a job takes unexpectedly long or short (e.g. when a burglary alarm turns out to be a false alarm).
- A time window $[e_i, l_i]$. The security company is contractually obliged to visit within this time frame. Most time windows have an interval of multiple hours, some less than an hour. An incident time window is either 30 or 45 min.
- A priority *p*, ranging from 1 to 4. 1 and 2 for incidents, 3 and 4 for static jobs, 1 being the highest priority, e.g. a fire alarm. Some customers have more expensive fees for tardiness and thus have a higher priority.
- An availability time or occurrence time. All static jobs are available at $t=0$. Incidents will become available during the day. The availability time of an incident is equal to its time window start time $e_i$, because incidents can always be visited as soon as they become available, in contrast to static jobs.

The jobs which are known a-priori will be referred to as static jobs. Static jobs have an average service time of 25 min, ranging from 1 min for a short check to 8 h for a surveillance. The dynamically assigned jobs are referred to as incidents. Incidents have an average service time of 16 and a half minute, but their total range is from only a few seconds (false alarm) up to multiple hours in case of a burglary arrest. However, usually an incident takes 10–30 min. Locations are usually clustered in business areas.

### Gaps and adaption

At the moment there is almost no dynamicity implemented in the baseline algorithm used in the business case. All jobs which are known a-priori, the static jobs, are scheduled by a state-of-the-art static VRPTW algorithm. The exact algorithm is unknown to us, as it is confidential. Also, a number of vehicles is always on stand-by. Their job is solely to react to any incoming incidents. Incidents are assigned by a (human) coordinator. In most cases an incident will go to the closest stand-by vehicle. In very rare cases, an incident will be picked up by a static job vehicle. The coordinator might need to do some manual rescheduling in this case.

This approach has some disadvantages:

1. The response to incidents might be too late if all incident vehicles are busy at the same time.
2. It takes time for the coordinator to plan all the incidents. Especially when multiple incidents come in at once and routes need to be rescheduled.
3. On a quiet day (a day with less than average incidents), the incident vehicles will be idle most of the time. This results in unnecessary labor time and bored employees.

Possible advantages of such an approach are:

1. Static job vehicle drivers know exactly what they have to do all day. This can make them more efficient and/or confident.
2. Incident vehicle drivers can specialize themselves in handling incidents. Training costs could be cheaper as apposed to a dynamic solution where all employees should be able to respond to any type of customer.

**Fig. 3:**
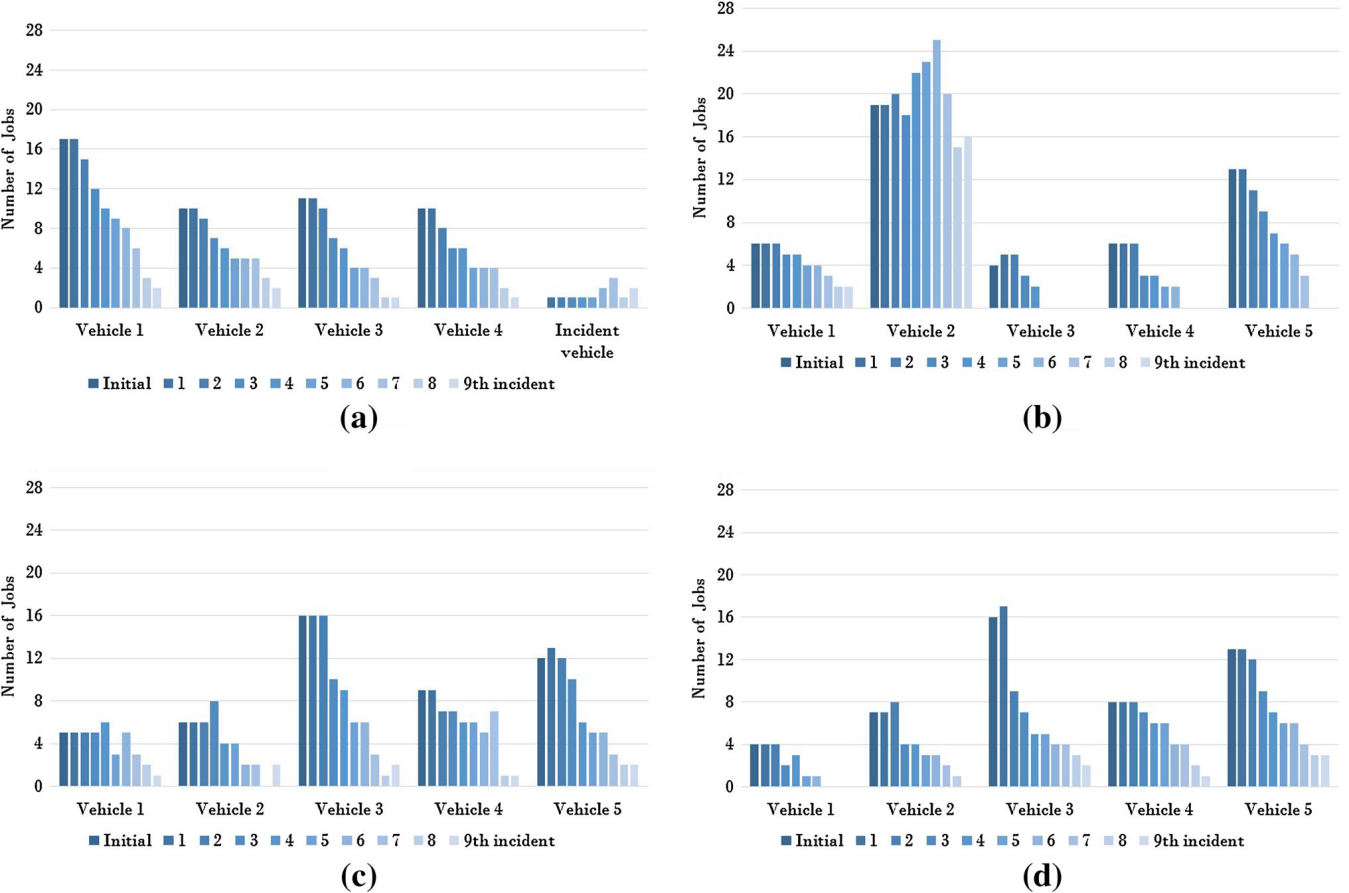
The total amount of jobs during **a** Pilot 1-Team A, **b** Pilot 1-Team B, **c** Pilot 2-Team C and **d** Pilot *S*-Team D. The *vertical axis* shows the number of orders that need to be served. For each vehicle, this is plotted for the times that a new incident occurred

In order to test the MACS algorithm, trail 1 is implemented to find the gaps between the theory benchmark problem and the real-world problem. The conclusions drawn from the first pilot were used to improve the implementation of the algorithm. A list was made of each required improvement and these were implemented iteratively. The most important revisions were:

1. Balancing of the vehicles. During the pilot some vehicles were very busy, while others had hardly any work (i.e. 25 and 2 jobs respectively). This can be seen in the results section, (Sect. 7) where Fig. 3b shows a vehicle with a significantly high amount of orders during the entire pilot. This fact resulted in the busy vehicles being late. Balancing also helps to give some buffer time, in case an incident has to be handled. Balancing was achieved by giving the vehicles a maximum amount of orders during initialization in the nearest neighbor algorithm. This maximum was chosen as $n/(n_v-1)$, where $n_v$ is the maximum of vehicles can be used in the pilot.
2. When a driver is already performing a job or driving towards a job, he/she should not be interrupted. I.e. this job should not be reassigned to another driver.
3. At the moment of recalculating the routes, it is important to keep track of the current time and the current position of the vehicles to check if any vehicles will be late. It might be necessary to reschedule in order to prevent tardiness.
4. The vehicle speed used in planning was assumed too high initially, since most of the pilot took place in an urban area. It was reduced to 30 km/h.

Also the controller was changed to be adjusted to the real-world situation. The controller of the implemented algorithm is displayed in Algorithm 6. The adjustments to this controller are:

1. The algorithm is not constantly searching for better routes. This is because the amount of changes to driver schedules should be minimized to avoid confusing the drivers. The cost of a small change would possibly be greater than its gain.The algorithm is not actively calculating after updating the schedules and before a new incident is introduced.
2. The number of iterations used by the ant colonies was set to 5000. This number was found to produce acceptable results within a minute. A short total calculation time was necessary to update routes as quickly as possible after an incident occurred. This number might need to be changed when the test case is scaled up or down.
3. The first job of a vehicle will always be locked on the first position of its route. This is so the driver never loses a job he/she is already performing. Also, when a driver started driving towards a customer, this customer should not be rescheduled to another driver.

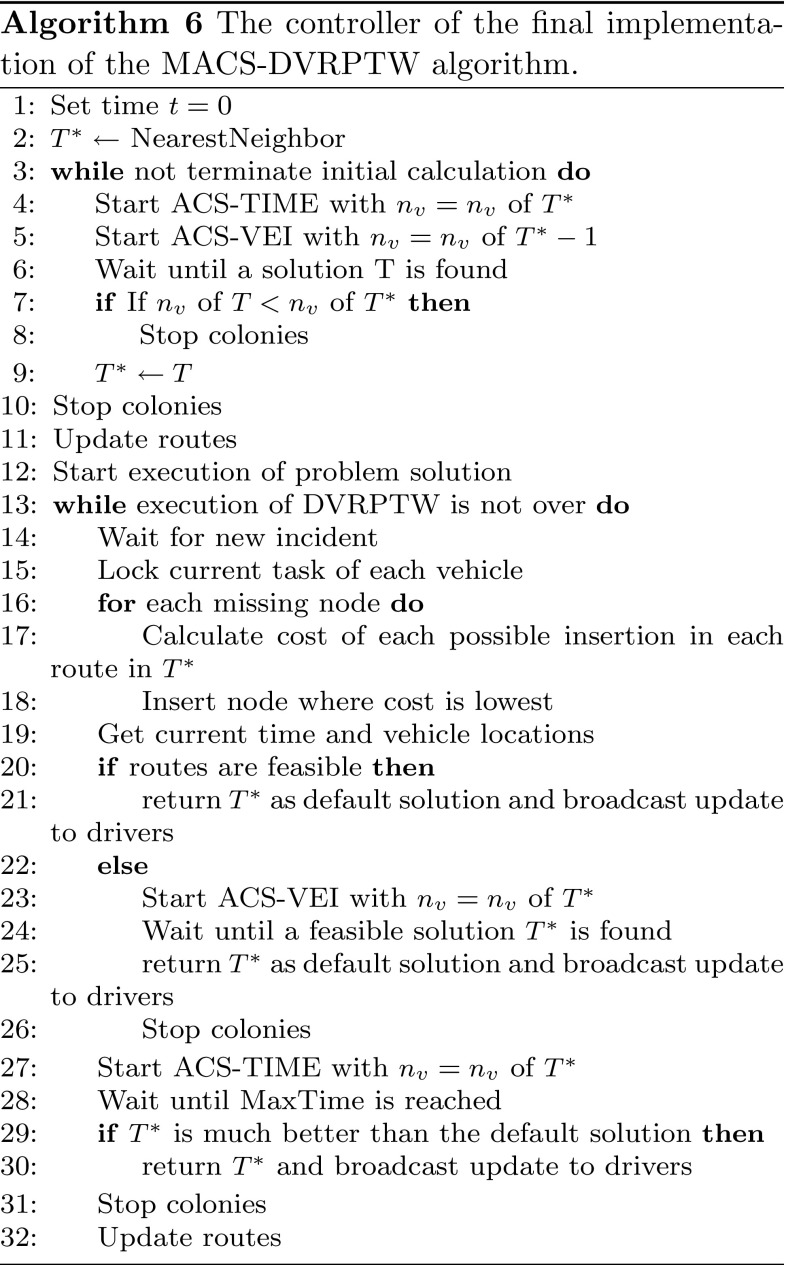

Other important adjustments to the algorithm were:

1. High priority is given to returning as fast as possible a feasible solution. This is why directly after finishing the direct insertion method already a solution can be returned to the controller; If there is no feasible solution available ACS-VEI is used first, as it searches with priority for feasible solutions.
2. ACS-TIME is used to find improvements of feasible solutions after having found a default feasible solution. Only if it succeeds to find a much better solution (a threshold is used here) this new solution will be returned and broadcast as an update to the drivers.
3. If the colony is trying to add missing nodes to an infeasible route, the highest priorities will be added first, if possible. The missing nodes are sorted by priority.
4. Feasibility of a route is based on the current location of the vehicles, which can be viewed as starting positions or depots when introducing an incident. Feasibility is also based on the time at the moment of calculation. Therefore, past time windows will not be considered anymore. By considering time and vehicle locations, more accurate schedules can be made when introducing a new incident while vehicles are driving towards a job. The feasibility check is based on the time and location which are retrieved.
5. Driving speed is by default 30 km/h, which is a good average speed for urban areas, allowing for some buffer time. Also in many areas the max speed is 30 km/h by law.
6. The nearest neighbor heuristic intends to distribute the jobs relatively even across the vehicles. This will give a balanced initial solution for the ACO pheromone initialization. Recall that, this is achieved by giving each vehicle a maximum of $n/(n_v-1)$ jobs.

### Pilot experiments

Next, the practical details of the experiments and the observations that were made will be discussed. To successfully implement a DVRP it is crucial to know the location of the vehicles and their status at the moment of occurrence of a new job. To achieve this, the DEAL platform which can be seen in Mahr and de Weerdt (2005) was used. This platform is made for managing workflows in logistics. All drivers can use a mobile application to update their status and GPS locations. The DEAL mobile application also shows to the drivers and the coordinators the sequence of jobs and their locations. The ACO algorithm was implemented as an external algorithm agent which was able to get an overview of the available jobs and the available vehicles. When this algorithm agent was triggered, it used ACO to rearrange the routes of the vehicles.

To test how well the algorithm performed in practice, two teams with five drivers each were hired. Team *A* worked according to the solution of the baseline algorithm provided by the security company. For this team four cars were assigned to static orders in a predetermined schedule, while one car visited all the incidents. It was used as a control group for baseline comparison. While Team *B* tested the performance of the MACS algorithm. All five cars were assigned to the static orders. When a new incident occurred, it would be assigned to one of these running cars based on the algorithm. In order to get a fair comparison between teams, both teams got their jobs assigned to them through the DEAL mobile application. However, Team *A*’s incident driver got a text message each time he or she was assigned to the new incident as common practice for the security company. Team *B*’s drivers were instructed to be aware of changing routes at all times. Each time an incident became available, the agent was triggered to change Team *B*’s routes. This was done on-the-fly. Both team started by the time that would enable them to reach their first address on time, according to the security company’s planning. Team *B*’s vehicles all were available for incidents from the time that they started.

The second pilot experiment consisted of only five drivers, referred to as Team *C*. This pilot became necessary because of shortcomings in the new scheduling method that needed to be corrected. For reasons of cost and practical feasibility another control group was not included. The first control group results proved very consistent and there was no strong need to test these results again, since the situation was expected to be very similar. Both pilots were conducted on a Friday, during the same time period, with no large weather differences. However, a small bias was introduced by an unexpected traffic jam that occurred during the second pilot. Much like Team *B* of Pilot 1, the five cars of Team *C* were sent out to visit their dynamic routes, which were determined on-the-fly by the (improved) algorithm agent. This time, there was a bigger focus on the minimization of labor hours, therefore not all cars started at the beginning of the pilot. Two cars started driving at the start of the pilot. Three other cars were given a customized starting time, based on the start of the time window of their first planned job.

As mentioned above, during Pilot 2, a traffic jam occurred which made some orders late and some orders failed. Because another pilot was not affordable, we decided to make a virtual Team *D* to do a simulation pilot (Pilot *S*) based on the data obtained in Pilot 2.

## Results

This section contains and discusses the results of all conducted pilots and of the simulated Team *D*. First of all, the performance of the teams will be discussed. After that, the survey of the drivers’ experience will be summarized. Finally, the lessons learned on bridging theory and practice will be summarized in order to help other researchers to implement their algorithm in the real world.

### Performance assessment

All the data during the pilots was stored which gave us a good insight into the real-world timing of the algorithm. For MACS, to perform well on the business case, it is important that there are as little contract violations as possible. Therefore, it is important to look at the timeliness of drivers, since they could arrive too late. It is also possible that a job is not visited at all, either because the driver was running too late or because the algorithm saw this as infeasible. In a very rare occasion (twice) the job was started before the time window, this is (in our case) due to human error.

The static jobs for Team *A* (Control Group in Pilot 1), Team *B* (Pilot 1), Team *C* (Pilot 2) and Team *D* (Simulation Group in Pilot *S*)are shown in Table 5. And in Table 6 the incident results can be seen. These results show us that the control group performed relatively well and stable. No control group driver arrived too late for either a static event nor for an incident. The route which was executed by the control group was based on the planning of the security company. The company executed this route many times before the pilot ran.

**Table 5:** The timeliness of the 48 static jobs

Static jobs				
	Pilot 1	Pilot 1	Pilot 2	Pilot *S*
	Team A	Team B	Team C	Team D
Not visited (#)	0	16	2	0
Not visited (% of total)	0	33.33	4.17	0
Late (#)	0	6	5	0
Late (% of finished)	0	18.75	10.87	0
Late (min)	0	106	50	0
Too early (#)	1	0	1	0
Too early (min)	8	0	3	0

**Table 6:** The timeliness of the nine incidents for both pilots

Incidents				
	Pilot 1	Pilot 1	Pilot 2	Pilot *S*
	Team A	Team B	Team C	Team D
Not visited (#)	0	4	0	0
Not visited (% of total)	0	44.44	0	0
Late (#)	0	1	1	0
Late (% of finished)	0	20	11.11	0
Late (min)	0	57	2	0

The first algorithm pilot experienced some problems. The most important problems are mentioned in Sect. 6.2, since they were used to improve the implementation before starting Pilot 2. The problems in Pilot 1 caused a significant amount of jobs to fail or at least be late. This can be seen in both Tables 5 and 6. More than one third of the jobs were not finished in Pilot 1. This is not acceptable for the business case. An important cause of this tardiness was the fact that one vehicle was scheduled to have more jobs than it could handle. Figure 3b shows that vehicle 2 was given much more orders than the other vehicles. This problem remained during the entire pilot, even though vehicle 3 was already finished with its jobs by the time the fifth incident occurred. This vehicle could have taken on some of the excess jobs from vehicle 2, but it didn’t.

After making the improvements of Sect. 6.2, Pilot 2 was conducted. A great improvement compared to Pilot 1 was observed. In Fig. 3c we can see that the jobs are more evenly distributed between vehicles and that these total amounts have a downward slope as time progresses.

Partly because of this even distribution, the timeliness of Pilot 2 was a lot more acceptable. Only 2 (static) jobs remained unvisited. Five jobs were too late with a total late time of 50 min. However, halfway through the pilot, one of the drivers got stuck in the traffic jam which was not present during the control group pilot. Two jobs were located in the middle of this traffic jam, both with an arrival time relatively close to the planned arrival times of the control group (within the same hour). So it is safe to say that the control group could also have experienced some delay. Or at the very least we could say that the Pilot 2 driver would have experienced less or no delay if the traffic jam would have not been present.

In the Pilot *S*, there is no traffic jam any more. The results showed that all the jobs were visited and there were no late nor early jobs. With this, we have more evidence that the algorithm can succeed in practice, under normal circumstances.

For the real-world case, the most important metric is the total labor time. These results are presented in Table 7. The total labor time needed would be the accumulated driving times of all cars, including driving from and towards the depot. The total driving times without driving times to and from the depots are also shown. This provides an impression of the on-line performance, excluding the influence of the starting and finalization strategy. The total time of Team *B* and Team *C* seem to be the shortest, but this is because jobs were left unfinished. For Team *D* we see an reduction in total labor time of 5 % compared to the control group.

**Table 7:** The total driving times, or labor hours, for both pilots and simulated pilot

Total driving time in hours				
	Pilot 1	Pilot 1	Pilot 2	Pilot *S*
	Team A	Team B	Team C	Team D
Excluding depot	25:59	19:57	22:57	**24:41**
Including depot	27:26	21:33	25:05	**26:09**

### Drivers experience survey

During this pilot the drivers took some forms with them so that they could take notes about their jobs, including arrival times and stress-levels. This was done to gather insights into the human factor of the implementation. The most important outcomes of the survey of Pilot 1 were:

1. The changing of routes was experienced as ‘confusing’ by some drivers.
2. A driver felt it was pointless that he had to drive back and forth from one side of the city to another side and back again. The experience of the driver was negative because he did not know the global solution.
3. Most stress was experienced by drivers that were running late.
4. Most drivers said they felt more confident about the execution of their tasks because they got a clear briefing beforehand and because they could contact a coordinator at all times.
5. Most drivers felt the planning was tight, but not too tight or stressful.

Outcomes 1 and 2 were only relevant for the drivers that tested the dynamic ACO algorithm (Pilot 1). From the survey of Pilot 2, also the outcomes 3 and 5 were found. Furthermore, the following results came out of the survey:

6. Two drivers found that a more frequent refresh of the job list would be helpful. A forced refresh each time a route is changed might even be more effective.
7. One driver experienced quite some stress during a traffic jam.
8. Four drivers already participated in the first trial, and experienced the second went much smoother. This was accounted mostly towards the relative absence of problems, such as disappearing jobs.

The drivers of Pilot 2 were given a form to write down their arrival times and also their stress, confidence, or certainty level. Ranges are from 1 to 5, were 1 is ‘(almost) none’ and 5 is ‘a lot’. Stress and confidence level where evaluated when arriving at a job.

At most times (42/55) stress was 1 (very low) and confidence was 5 (very high). When stress went up, that usually meant that the driver’s confidence was low. (7/12) The drivers experienced stress in the following occasions:

- The driver was running late.
- The driver got stuck in traffic.
- The driver took a wrong turn, delaying his route.
- The driver was not sure if finishing a job outside of the time window also counted as being late.

The first and the second situations can (partly) be reduced in their number by making smart algorithms and adding data on traffic situation. For avoiding the third situation, training of the drivers and inclusion of buffer time could be beneficial. The last situation can be easily avoided by a better briefing of the drivers.

### From theory to practice: lessons learned

Implementing in practice means testing in practice. When working with real-world cases and data, one cannot simply implement something and only test on academic benchmarks. Some general lessons on bringing routing algorithms from theory to practice have been learned and we condensed them to three key principles:

- *Iteration works* It is impossible to know all the functionality of the algorithm implementation and situations that might occur in practice beforehand. Therefore it is important to keep in mind that requirements might change. A real-world test will give a clearer look on the elements needed. It is however still a good idea to get a head-start on the requirements by doing simulated benchmarks. Starting with a thorough analysis of the business case can also give a good indication of what particularities require attention. In our first pilot, we could have avoided some mistakes by better analyzing the effect of clustering on the job distribution. Handling of various kinds of constraints is often specific to the real-world scenario and algorithms will only succeed if they are flexible enough for adaptation.
- *Communication is key* Implementing an algorithm in a real-world environment is not a one man’s job. In our case we needed at least an optimization algorithm expert, a logistics systems/workflow manager (DEAL), a logistics company providing a business case, and a team of drivers. These experts had to be able to communicate with each other. Social aspects of the project as well as business aspects needed to be addressed, besides technical aspects. While confidentiality issues needed to be respected, at the same time it was to be made sure that enough insights were gained from the pilot in order to improve algorithmic methods.
- *People are important* The customers and drivers should play an important role in the development of the end results. After all, they will be using it and if they don’t understand the algorithm’s instructions they may even start to ignore them or complain. We found that a clear briefing and description of tasks and expectations contributed to the confidence of the drivers. Changing of routes comes at a psychological cost, as the driver was already primed (mentally prepared) for another task. Therefore, the changing of routes should be presented as transparent as possible so the employee comprehends the logic of his route sufficiently, i.e. does not doubt the efficiency of the schedule. It is also important to consider that an employee needs to feel useful and needs to have the feeling that he/she is treated fair.

## Summary and outlook

This work proposed a dynamic algorithm for VRPTW that allows to integrate new orders during operation in a schedule. A new algorithm, MACS-DVRPTW, was introduced and described. It is an extension of the state-of-the-art ant colony based meta-heuristic MACS-VRPTW for dynamic VRPTW problems. A dynamic benchmark is created based on the static Solomon’s benchmark for VRPTW, by revealing some of the orders only during operation time to the algorithm. Statistical studies were conducted, showing that MACS-DVRPTW algorithm performs better than the state of the art algorithms on the academic benchmarks. In the pilot experiments adaptations were needed in order to achieve competitive performance. The new version of the algorithm performs better than the solution by the company in terms of total driving time, but it requires still improvement in terms of real-world constraint handling for special situations such as traffic jams. And it will also be interesting to compare this algorithm with other dynamic methods such as Wang et al. (2010), Lung and Dumitrescu (2010).

Another major finding was that the human factor is important. In order to account for this in the development phase, three main principles have crystallized out that we summarize as: iteration works, communication is key, and people are important.

In future work these principles need to be more fully used. Besides optimization also the interaction between drivers and software seems to play a major role. Here techniques from transaction management could prove to be useful, e.g. to design a protocol that makes it possible to deal with sudden changes of the situation such as traffic jams and makes regular checks on the feasibility of the current plan based feedback on the drivers location. A full integration of the available information from GPS tracking will however require major adaptation to the design of scheduling algorithm and it will therefore be left for future work.
